# Pharmacological Targeting of Microglial Activation: New Therapeutic Approach

**DOI:** 10.3389/fncel.2019.00514

**Published:** 2019-11-19

**Authors:** Cai-Yun Liu, Xu Wang, Chang Liu, Hong-Liang Zhang

**Affiliations:** ^1^Department of Neurology, The First Hospital of Jilin University, Changchun, China; ^2^Department of Life Sciences, National Natural Science Foundation of China, Beijing, China

**Keywords:** Parkinson’s disease, microglia, neuroinflammation, microglial activation, polarization

## Abstract

Mounting evidence suggests that neuroinflammation is not just a consequence but a vital contributor to the development and progression of Parkinson’s disease (PD). Microglia in particular, may contribute to the induction and modulation of inflammation in PD. Upon stimulation, microglia convert into activated phenotypes, which exist along a dynamic continuum and bear different immune properties depending on the disease stage and severity. Activated microglia release various factors involved in neuroinflammation, such as cytokines, chemokines, growth factors, reactive oxygen species (ROS), reactive nitrogen species (RNS), and prostaglandins (PGs). Further, activated microglia interact with other cell types (e.g., neurons, astrocytes and mast cells) and are closely associated with α-synuclein (α-syn) pathophysiology and iron homeostasis disturbance. Taken together, microglial activation and microglia-mediated inflammatory responses play essential roles in the pathogenesis of PD and elucidation of the complexity and imbalance of microglial activation may shed light on novel therapeutic approaches for PD.

## Generic View of PD Pathogenesis

Parkinson’s disease, a prevalent movement disorder which affects almost 2% of the aged population worldwide, is the second most common neurodegenerative disease (Hirsch et al., 2016). Pathologically, PD is characterized by the selective and progressive degeneration of dopaminergic neurons in the SNpc and dopaminergic terminals in the striatum. The exact molecular mechanisms of neuronal loss are not fully understood, but several pathways and mechanisms are known to be implicated in PD pathophysiology, including neuroinflammation, impairment of autophagy, oxidative stress, severe endoplasmic reticulum stress, mitochondrial dysfunction, dysfunction of axonal transport, motor circuit pathophysiology, α-syn aggregation, and prion-like cell-to-cell transmission of α-syn (Charvin et al., 2018). Although it may not be the initial trigger of PD, neuroinflammation is considered to be a vital promoter to the development of PD, in which microglia perform significant roles (Hirsch and Hunot, 2009; Wang et al., 2015c; Skaper et al., 2018).

McGeer et al. (1988) first proposed that innate immunity was involved in PD in 1988 when a substantial number of HLA-DR-positive reactive microglia were detected in the SN of brains from post-mortem PD patients using immunohistochemical staining. Another neuropathologic study showed that the number of reactive microglia expressing MHC class II molecules not only in the SN and putamen but also in the hippocampus, cingulate cortex, temporal cortex and transentorhinal cortex was increased as the neuronal degeneration proceeded (Imamura et al., 2003). Similar up-regulated expression of MHC, as well as T-cell infiltration, has been reported in the SN and striatum of MPTP mouse model of PD (Kurkowska-Jastrzebska et al., 1999). Moreover, GWAS further indicated that common genetic variation in the HLA region was related to sporadic PD (Hamza et al., 2010; Hill-Burns et al., 2011; Nalls et al., 2011). *In vivo* studies using PET confirmed that microglia were widespread activated at the early stage of PD and levels remained relatively stable, possibly driving the disease process via inflammatory reaction (Ouchi et al., 2005; Gerhard et al., 2006; Edison et al., 2013; Iannaccone et al., 2013; Femminella et al., 2016; Ghadery et al., 2017).

The brain of PD patients and animal models induced by MPTP or 6-OHDA showed several signs of increased inflammatory reaction and programmed cell death (or apoptosis) of neurons and/or neuroglia (Nagatsu et al., 2000; Hunot and Hirsch, 2003): (a) microgliosis which was observerd in various toxin-based models, such as MPTP, 6-OHDA, and rotenone (Marinova-Mutafchieva et al., 2009; Fricke et al., 2016; Ojha et al., 2016; Manocha et al., 2017), as well as in mutant α-syn transgenic models of PD (Lee et al., 2002; Su et al., 2009); (b) elevated levels of inflammatory cytokines (Mogi et al., 1994a, b, 1996), such as IL-1β, IL-2, IL-4, IL-6, IFN-γ and TNF-α, TGF-α, TGF-β1, TGF-β2, EGF, bFGF; (c) increased levels of cytokine receptors, such as TNF-α receptor R1 (p55); (d) increased levels of caspase activities, such as caspase-1 and caspase-3; (e) enzymes related to inflammation (Knott et al., 2000), such as COX-1, COX-2, and iNOS (Knott et al., 2000; Iravani et al., 2002; Ruano et al., 2006); (f) reduced levels of neurotrophins, such as NGF and BDNF; (g) elevated levels of bcl-2 and soluble Fas, which could protect and promote apoptosis, respectively.

A remarkable percentage of monocytic precursors were found in the peripheral blood of patients with PD (Funk et al., 2013). Moreover, increased effector/memory T cells (Tem), defined as CD45RO^+^ and FAS^+^ CD4^+^ T cells, and decreased CD31^+^ and α4β7^+^ CD4^+^ T cells were associated with progressive Unified PD Rating Scale III (UPDRS-III) scores (Saunders et al., 2012). Collectively, the immune responses, notably the CD4^+^-cell sub-set imbalance and Tem activation, may mirror the pathobiology of PD in the CNS.

Several meta-analyses revealed the association between the use of NSAIDs and the risk of PD (Samii et al., 2009; Gagne and Power, 2010; Gao et al., 2011; Rees et al., 2011; Poly et al., 2019), whereas the relationship was inconclusive. NSAIDs as a whole seemed not to be related to the risk of PD (Samii et al., 2009; Poly et al., 2019). Interestingly, subgroup analysis indicated that use of nonaspirin NSAIDs may have a protective effect, though not shared by aspirin (Gagne and Power, 2010; Rees et al., 2011) or acetaminophen (Gagne and Power, 2010; Gao et al., 2011). One prospective study suggested that the use of nonaspirin NSAIDs may reduce PD risk in males but not in females (Hernan et al., 2006). One population-based study reported that the effect of aspirin also differed by gender, namely a protective role only in females, especially for longer duration of use (≥24 months) or at higher doses (≥14 pills/week) (Wahner et al., 2007). This gender-specific association between NSAIDs and lower PD risk warrants further investigation. As regards ibuprofen, whether there is a potential therapeutic effect or not is still controversial (Chen et al., 2005; Samii et al., 2009; Gao et al., 2011). Further, in MPTP-induced mouse models, indomethacin exerted anti-inflammatory effects and promoted survival of new neurons in the hippocampus without reversing dopaminergic neuronal loss (Hain et al., 2018). Rofecoxib and ibuprofen were also shown to directly protect MPTP-induced damage (Gupta et al., 2009; Swiatkiewicz et al., 2013). In an *in vitro* study, 6-OHDA-induced PC12 cells pretreated with celecoxib, indomethacin and ibuprofen for 24 h showed significantly increased cell viability, GSH content, and cytoplasmic level of NF-κB, as well as decreased levels of ROS and several apoptosis biomarkers, including cleaved caspase-3, Bax, P-SAPK/JNK and cleaved PARP (Ramazani et al., 2019). Further studies are still needed to elucidate the underlying inflammation process in PD and possible protective effects of NSAIDs.

Taken together, neuroinflammation appears to play central roles in the development of PD. Glial cells, particularly microglia, are involved in this scenario.

## Generic View of Microglial Functions

Microglia are resident macrophage-like immune cells in the CNS (Lawson et al., 1990). “Resting” microglia exhibit a ramified morphology with elongated motile cytoplasmic protrusions and processes; they constantly survey the CNS microenvironment and dynamically interact with other neighboring elements, such as neuronal cell bodies, astrocytes, and blood vessels (Davalos et al., 2005; Nimmerjahn et al., 2005; Szalay et al., 2016; Catalin et al., 2017).

**FIGURE 1:**
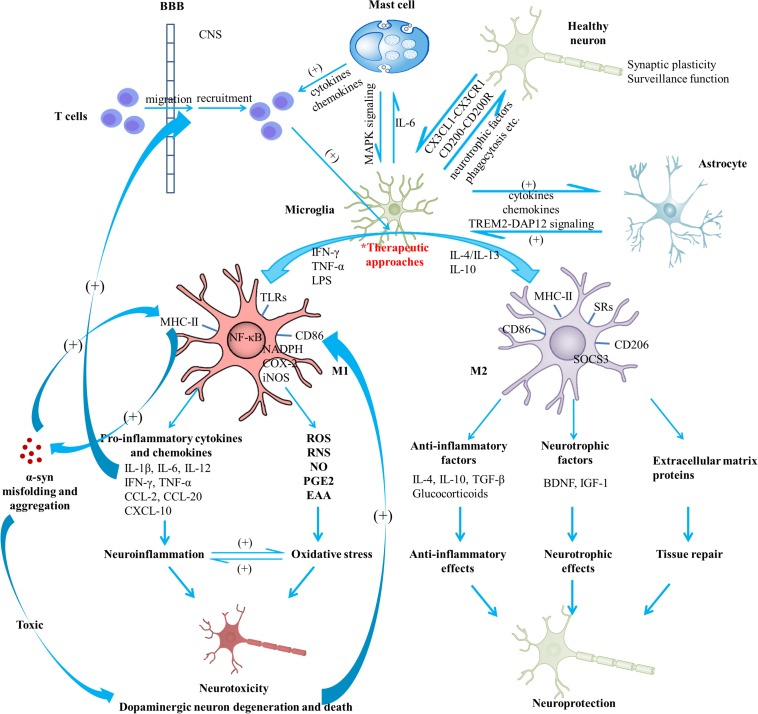
Schematic view of microglial activation and cross-talks between microglia and other immune factors in the pathogenesis of Parkinson’s disease. With regard to PD pathogenesis, microglial activation and microglia-mediated inflammatory responses play essential roles. The M1 polarized state can be induced by TNF-α, IFN-γ, and LPS; present phenotype markers such as MHC-II and CD86; are associated with the production of pro-inflammatory cytokines (IL-1β, IL-6, IL-12, IFN-γ, and TNF-α), chemokines (CCL-2, CCL-20) and CXCL-10; are capable of releasing cytotoxic substances (ROS, RNS, NO, EAA) and PGE2 due to the activation of NADPH oxidase, iNOS, as well as expression of COX-2, contributing to the enhanced oxidative stress. Neuroinflammation and oxidative stress interact with each other to engender a vicious cycle, exerting toxic effects on dopaminergic neurons and leading to the exacerbation of the neurodegenerative process. On the other hand, M2 can be induced by IL-4, IL-10, and IL-13; generally present intracellular components (e.g., SOCS3), cell surface markers (SRs, CD206, CD86, and MHC-II); are capable of producing mediators such as anti-inflammatory cytokines (e.g., IL-4, IL-10, and TGF-β), glucocorticoids, neurotrophic factors (e.g., BDNF and IGF-1), and extracellular matrix proteins; exert anti-inflammatory and neurotrophic effects and play vital roles in tissue repair. Notably, activated microglia may exist along a dynamic continuum rather than be simply polarized into two categories, which is regulated by interactions with other cellular elements, including astrocytes, neurons, infiltrating T cells and mast cells. TREM2-DAP12 signaling complex, the CD200-CD200R and the CX3CL1-CX3CR1 axes are considered to be involved in the astrocyte-microglia and neuron-glia cross-talks. Activated mast cells could induce microglial activation via the MAPK signaling pathway. In turn, microglia-derived IL-6 could induce surface TLR2 and TLR4 expression and consequent cytokine release of mast cells, which contribute to the recruitment of immune cells to the injured areas. In addition, microglial activation has been indicated to promote the prion-like behavior of α-syn misfolding and aggregation. In turn, misfolded α-syn act as chemoattractants to direct microglial migration toward damaged neurons, promote the pro-inflammatory microglia, and exert toxic effects on neurons. Dopaminergic neurons in a death process can trigger microglial activation and inflammatory factor production which can promote recruitment of peripheral leucocytes (mainly T cells). In this way, a complex inflammatory network forms and aggravates degeneration of dopaminergic neurons. A variety of agents or approaches might exert neuroprotective effects due to their regulatory roles on microglial activation. BBB, blood-brain barrier; BDNF, brain-derived neurotrophic factor; COX, cyclooxygenase; CXCL, chemokine (C-X-C motif) ligand; DAP12, DNAX activation protein 12; EAA, excitatory amino acids; IFN, interferon; IGF-1, insulin-like growth factor-1; IL, interleukin; iNOS, inducible nitric oxide synthase; LPS, lipopolysaccharide; MAPK, mitogen-activated protein kinase; NADPH, nicotinamide adenine dinucleotide phosphate; NO, nitric oxide; PD, Parkinson’s disease; PGE2, prostaglandin E2; RNS, reactive nitrogen species; ROS, reactive oxygen species; SOCS3, suppressor of cytokine signaling 3; SR, scavenger receptor; TGF, transforming growth factor; TLR, toll-like receptor; TNF, tumor necrosis factor; TREM2, triggering receptor expressed on myeloid cells 2; α-syn, α-synuclein.

Microglia actively contribute to brain development, injury repair, as well as homeostasis maintenance and surveillance (Michell-Robinson et al., 2015; Colonna and Butovsky, 2017). In the developing CNS, microglia control its patterning by regulating apoptosis of neuronal subpopulations (Wakselman et al., 2008) as well as neuron survival and proliferation (Ueno et al., 2013), and control the wiring of neurons by modulating synapse function and maturation, activity-dependent synaptic pruning, as well as synapse number (Wake et al., 2009; Paolicelli et al., 2011; Schafer et al., 2012; Kierdorf and Prinz, 2017). Acting similarly to peripheral macrophages, microglia in the mature CNS are able to phagocytize excess synapses, dead cells, protein aggregates, pathogens, and other particulate and dissolvable antigens that may endanger the CNS (Neumann et al., 2009; Sierra et al., 2010; Kettenmann et al., 2011; Colonna and Butovsky, 2017). Moreover, microglia secrete cytokines, chemokines, growth factors, ROS and RNS, and PGs (Gonzalez et al., 2014). These functions of microglia can be regulated by interactions with the BBB and other cellular elements, including astrocytes, neurons, infiltrating T cells and mast cells (Gonzalez et al., 2014; Figure 1). Microglia show brain region-dependent diversity and selective regional sensitivities to aging, which could underlie region-specific sensitivities to microglial dysregulation and involvement in age-related neurodegeneration (Grabert et al., 2016).

## Polarization of Microglia and Its Roles in Neuroinflammation

In various neurodegenerative diseases or in response to any insult, microglia become activated, including morphological changes, alterations of gene expression and surface markers, and proliferation (Wolf et al., 2017). Activated microglia may present epithelioid, rod, amoeboid, multinucleated or “dystrophic” morphological appearance (Boche et al., 2013). Growing evidence shows that microglial activation in the CNS is heterogeneous, similarly to peripheral macrophages, which is often simplified into two polarization states: the M1 phenotype (known as “classical activation”) and the M2 phenotype (known as “alternative activation”) (Orihuela et al., 2016). Activation of microglia can be induced by various factors through numerous pattern recognition receptors like TLRs, SRs and cytokine receptors and chemokine receptors. Microglial cells may present distinctive phenotypes and exhibit specific effects depending on the nature, intensity and duration of the stimulus (Ransohoff and Perry, 2009; Gosselin et al., 2017; Joers et al., 2017; Figure 1).

The M1 polarized microglia with an IL-12^high^, IL-23^high^, IL-10^low^ phenotype, present markers such as MHC-II, COX-2, iNOS, and CD86 (Martinez et al., 2006; Chhor et al., 2013), which are associated with the production of pro-inflammatory cytokines (IL-1β, IL-6, IL-12, IFN-γ, and TNF-α), chemokines (CCL-2, CCL-20), CXCL-10, cytotoxic substances (ROS, RNS, NO, EAA), and prostaglandin E2 (PGE2) (Le et al., 2001; Mantovani et al., 2005; Pais et al., 2008). M1 microglia are able to promote neurogenesis and astrocytogenesis from NSPCs (Butovsky et al., 2006; Vay et al., 2018), participate as inducer and effector cells in polarized Th1 responses, and mediate resistance against tumor cells and intracellular parasites (Mantovani et al., 2005). This polarized phenotype can be induced in experimental animals by using IFN-γ or LPS. IFN-γ mediates M1 polarization of microglia mainly through the JAK/STAT intracellular signal transduction pathway; also by “priming”, enhancing TLR-activated signal transduction and then reinforcing microglial responsiveness to inflammatory stimuli (e.g., TLR ligands and TNF-α) (Hu and Ivashkiv, 2009). Studies *in vivo* and *in vitro* on macrophage showed that TRPC1-mediated Ca^2+^ influx is crucial for IFN-γ-induced polarization to the M1 phenotype (Chauhan et al., 2018). LPS, an endotoxin in the outer membrane of Gram-negative bacteria, induces M1 polarization via binding to TLR4, which is coupled to myeloid differentiation protein 2, with participation of LPS-binding protein and co-receptors CD14 (Chow et al., 1999; Takeda and Akira, 2004). The TLR4-mediated signaling typically involves an MyD88-dependent pathway, in which activation of transcription factor NF-κB is followed by production of several inflammatory cytokines (e.g., IL-1β, IL-6, TNF-α), and an MyD88-independent pathway, in which activation of transcription factors NF-κB and IRF-3 is followed by the production of IFN-β mediating IFN-β-induced STAT1-dependent gene expression (Chow et al., 1999; Kawai et al., 2001; Toshchakov et al., 2002; Takeda and Akira, 2004). In this way, expressions of numerous M1-associated cytokines, chemokines and other inflammatory factors are increased. These cytokines activate iNOS and NADPH oxidase, leading to the production of NO, ROS, and RNS (Bove and Perier, 2012; Zeng et al., 2012).

The mechanism of M2 polarization in microglia, as compared to macrophages, is much less clear. The characteristics of M2 microglia may parallel those of macrophages to some extent (Chhor et al., 2013; Miron et al., 2013). The M2 state in macrophages can be divided into three sub-classes named M2a, M2b, and M2c, respectively (Martinez and Gordon, 2014), which might be extrapolated to microglia. The M2 polarized microglia with an IL-12^low^, IL-23^low^, IL-10^high^ phenotype, generally present high levels of scavenger, mannose-type and galactose-type receptors (Mantovani et al., 2005), which are associated with the production of anti-inflammatory cytokines (e.g., IL-4, IL-10, and TGF-β), glucocorticoids, neurotrophic factors (e.g., BDNF and IGF-1), and extracellular matrix proteins (e.g., fibronectin) (Subramaniam and Federoff, 2017). M2 microglia are able to promote oligodendrogenesis and neurogenesis (especially oligodendrogenesis) from NSPCs via the PPAR-γ signaling pathway (Butovsky et al., 2006; Vay et al., 2018) and perform vital roles in various situations, such as immune regulation, inflammation inhibition and tissue repair (Michell-Robinson et al., 2015). The activation mechanisms of these three sub-classes as well as their functions are different. The M2a activation state, which performs phagocytosis, functions to suppress inflammation, and contributes to tissue repair, can be induced by IL-4 or IL-13 (Stein et al., 1992; Joers et al., 2017; Subramaniam and Federoff, 2017). IL-4 *per se* mediates M2a polarization through binding to various receptor pairs, then stimulating JAK1 or JAK3, activating STAT6 and resulting in expression of M2a-associated genes, such as intracellular components (e.g., SOCS3), cell surface markers (SRs and CD206), Ym1 (chitinase-like protein), Fizz1 (found in inflammatory zone) and IL-10 (Stein et al., 1992; Joers et al., 2017; Subramaniam and Federoff, 2017). The M2b polarized state, which is involved in inflammation regulation and selective phagocytosis, can be induced by agonists of TLRs and IL-1 receptors. TLRs become activated by fusing Fc gamma receptors, which bind to immunoglobulin G complexes, resulting in macrophage/microglia polarization to an M2b phenotype and then leading to the secretion of IL-10 and the production of cell surface markers (MHC-II, CD86) (Franco and Fernandez-Suarez, 2015). The M2c polarized state, which plays vital roles in immune regulation, matrix deposition and tissue remodeling, can be induced by IL-10 and glucocorticoid hormones (Subramaniam and Federoff, 2017). IL-10 can activate JAK1 through binding to IL-10 receptor 1 and 2 subunits, then resulting in the translocation of STAT3 to the nucleus, which consequently inhibits the production of M1-associated pro-inflammatory cytokines (Subramaniam and Federoff, 2017).

In general, the M1 phenotype is considered to be the first line of defense with pro-inflammatory roles, while the M2 phenotype to suppress inflammation and to promote tissue repair. Of note is that activated microglia may exist along a dynamic continuum and get different profiles depending on their surrounding microenvironment, rather than be simply polarized into either category (Figure 1).

As mentioned above, the over-simplified “polarization” designation of activated microglia is still controversial. Additional microglia phenotypes which do not align with the traditional binary classification have been discovered (Holtman et al., 2015; Szulzewsky et al., 2015; Morganti et al., 2016; Cho et al., 2019). Therefore, polarization of microglia or the continuum from M1 to M2 state has not been fully supported by experimental data; more importantly, the microglial polarization paradigm may impede rather than facilitate research progress as this schema tends to simplify data interpretation (Ransohoff, 2016). Current research is focused on the characterization of activated microglia for discovering new drug target.

## Activation of Microglia and Pathogenesis of PD, What Have We Learned?

The complexity and imbalance of microglial activation has become an area of intense focus, aiming to elucidate a potential mechanism of PD pathogenesis and to identify potential targets for anti-inflammatory treatment (Du et al., 2017).

### Microglial Activation and Associated Neuroinflammation and Oxidative Stress

Studies in PD patients, alongside with mounting data in experimental models of this disease, clearly indicate that microglial activation and microglia-mediated inflammation exert dual (beneficial/harmful) effects in PD pathophysiology, as discussed above. A variant in the TREM2 gene of microglia, p.R47H, is a risk factor for PD (Rayaprolu et al., 2013). In the MPTP-induced mouse models of progressive PD, the degeneration of dopaminergic neurons was associated with gradual polarization of microglia to an M1 (pro-inflammatory) phenotype (Pisanu et al., 2014), which could become even more obvious with age (Yao and Zhao, 2018). Furthermore, microglia seemed to present dynamically changing phenotypes depending on disease-stage, accounting for the dynamic changes of pro-inflammatory and anti-inflammatory cytokines (Pisanu et al., 2014; Joers et al., 2017).

Activated microglia proliferate and exhibit a stage- and region-specific secretion of cytokines and mediators (neurotoxic molecules including NO, ROS, RNS and pro-inflammatory cytokines), leading to progressive degeneration of dopaminergic neurons, while microglia in the SNpc are more abundant (approximately 4.5 times) than in other brain regions (Grabert et al., 2016; Lopez Gonzalez et al., 2016). In addition, dopaminergic neurons in the SNpc have decreased anti-oxidant potentials, while the redox reaction of DA could be enhanced by the intensified generation of ROS, resulting in the production of toxic DA metabolites (Whitton, 2007; Tansey and Goldberg, 2010; L’Episcopo et al., 2018). All these features render midbrain dopaminergic neurons more susceptible to oxidative/inflammatory attacks (Whitton, 2007; Tansey and Goldberg, 2010; L’Episcopo et al., 2018). Hence, neuroinflammatory mechanisms could contribute to neuronal degeneration via cascade events.

With regard to the mechanism(s) of dopaminergic neuron degeneration and death, non-cell-autonomous mechanisms might play vital roles as well (Hirsch and Hunot, 2009). Activation of glial cells (especially microglia), triggered by dopaminergic neurons in a death process, could induce inflammatory responses as well as oxidative stress and cytokine-receptor-mediated apoptosis, which in turn promote recruitment of peripheral leucocytes (mainly T cells) and lead to an inflammatory network around neuronal injury (Hirsch and Hunot, 2009). The non-cell-autonomous mechanism is considered to drive dopaminergic cell death and hence neurodegeneration in PD (Hirsch and Hunot, 2009). During microglial activation, activation of NADPH oxidase, iNOS, as well as expression of COX-2 results in the synthesis of high levels of ROS, RNS, NO, and PGE2, contributing to the enhanced oxidative stress, which not only exerts toxic effects on dopaminergic neurons but also further amplifies the pro-inflammatory microenvironment (Taylor et al., 2013). Peroxynitrite, a potent toxin produced when NADPH oxidase and iNOS are present together, promotes the nitration of proteins (e.g., tyrosine) and further production of hydroxyl radicals, which can damage lipids, proteins, DNA and RNA, resulting in dysfunction and eventual death of neurons (Gao and Hong, 2008; Taylor et al., 2013; L’Episcopo et al., 2018). In this way, neuroinflammation and oxidative stress interact to engender a vicious cycle, leading to the exacerbation of neurodegeneration (Figure 1). However, microgliosis and increase of pro-inflammatory molecules could be detected at the early stage and well before dopaminergic neuron death in the α-syn transgenic models (Su et al., 2009), implying that cell death is not necessarily a prerequisite for the activation of microglia and mutated α-syn could also initiate the vicious cycle.

The interaction between α-syn and microglia is considered to be vital in the pathogenesis of PD (Figure 1). α-syn, a presynaptic protein secreted by neurons in small amounts which prevalently exists as monomers in physiology, is over-expressed and aggregates into oligomers or protofibrils in PD (Theillet et al., 2016). Oligomers/protofibrils could propagate between cells as free floating proteins or via extracellular vesicles (Li et al., 2008; Ingelsson, 2016), exert toxic effects on the synapses, resulting in disruption of electrophysiological properties (Calo et al., 2016; Ingelsson, 2016; Bridi and Hirth, 2018; La Vitola et al., 2018; Prots et al., 2018; Longhena et al., 2019), and act as chemoattractants to direct microglial migration toward damaged neurons (Wang et al., 2015a). In addition, α-syn over-expression promotes the polarization of microglia to the pro-inflammatory phenotype with elevated production of cytokines such as IL-1β, IL-6, and TNF-α as well as COX-2, iNOS and free radicals (Zhang et al., 2005, 2017; Austin et al., 2006; Su et al., 2008, 2009; Theodore et al., 2008; Sacino et al., 2014; Ferreira and Romero-Ramos, 2018). Oligomeric α-syn induces the M1 phenotype by directly engaging the TLR1/2 heterodimer, resulting in the nuclear translocation of NF-κB and the elevated production of these cytokines by an MyD88-dependent pathway (Kim et al., 2013; Daniele et al., 2015; Ho, 2019; Kouli et al., 2019): After α-syn activates TLR1/2 heterodimer, the C-terminal intracellular TIR domains of TLR interact with MyD88, activating the IRAK complex (Lin et al., 2010), which in return activates the TRAF6 via its K63-linked auto-ubiquitination. Then, the subsequent activation of the TAK1 complex and its downstream effector IKK complex lead to the degradation of IκBα and the production of pro-inflammatory cytokines via the activation of MAPK and translocation of NF-κB, p38 and JNK (Symons et al., 2006; Kawai and Akira, 2007, 2010). TLR4 could also mediate α-syn-dependent activation of microglia, including the production of ROS and pro-inflammatory cytokines as well as the phagocytic activity of microglia (Fellner et al., 2013; Janda et al., 2018; Hughes et al., 2019). Scavenger receptor CD36 and P2X7 receptor are also involved in α-syn-mediated microglial activation (Su et al., 2008; Jiang et al., 2015). Moreover, α-syn-stimulated microglia secrete MMPs, activating PAR-1 and amplifying microglial inflammatory signals (Lee et al., 2010). In this way, α-syn is a vital regulator of microglial activation and phagocytosis. Activated microglia which exert enhanced phagocytosis of α-syn in turn, could on one hand directly play a beneficial role in controlling α-syn spread (Stefanova et al., 2011), and on the other hand, notably, promote the prion-like behavior of α-syn misfolding, aggregation and spread (Hansen et al., 2011; George et al., 2019; Olanow et al., 2019). This may be associated with the increased accumulation of intracellular α-syn in microglia and accelerated secretion of α-syn into extracellular space via exosomal pathways (Porro et al., 2019; Xia et al., 2019), as well as the toxic effects of cytokines generated from activated microglia on the vulnerable neuronal cells (Olanow et al., 2019). Taken together, microglia-derived oxidative stress is considered to bridge α-syn pathogenic alteration and neuroinflammation, contributing to chronic progression of neurodegeneration in PD (Lawand et al., 2015). Those mentioned receptors on microglia could be therapeutic targets for microglial activation and α-syn pathogenesis.

### Cross-Talks Between Microglia and Other Cell Types

The microglial polarization status and associated neuroinflammation are tightly connected with astrocyte-microglia and neuron-glia interactions via ligand-receptor pairings (Figure 1). TREM2, exclusively expressed by microglia, could enhance the phagocytosis function of microglia (Takahashi et al., 2005, 2007; Kawabori et al., 2015), and attenuate inflammatory responses by negatively regulating the TLR4-mediated activation of NF-κB signaling and shifting M1 polarization to the M2 phenotype (Jiang et al., 2016; Ren et al., 2018; Zhang et al., 2018). In addition, TREM2-DAP12 signaling complex exerts vital roles in promoting phagocytosis, microglial activation, as well as microglial survival (Belloli et al., 2017; Konishi and Kiyama, 2018; Mecca et al., 2018). The fractalkine receptor CX3CR1, another receptor specifically expressed in microglia, plays a fundamental role in the communication between microglia and neurons by binding to neuronal fractalkine, namely CX3CL1 (Harrison et al., 1998). Accumulating evidence suggests that the CX3CL1-CX3CR1 axis is involved in homeostatic suppression of microglial activation and regulation of chemoattraction and synaptic plasticity, inhibiting microglia-mediated inflammatory responses and neurotoxicity (Cardona et al., 2006; Pabon et al., 2011; Sheridan and Murphy, 2013; Mecca et al., 2018). CD200, a transmembrane glycoprotein present on neurons, can regulate microglial activation status by binding to microglial CD200R. Deficits in the CD200-CD200R axis could exacerbate activation of microglia and dopaminergic neurodegeneration (Wang et al., 2011; Zhang et al., 2011).

Notably, the mast cell-glia cross-talk is also involved in regulation of neuroinflammation (Skaper et al., 2017, 2018; Figure 1). In inflammatory tissues, the expression of ligand-receptor pairings is increased, enhancing chemotactic actions of glial cells and mast cells and facilitating intercellular communication. Activated mast cells could induce microglial activation and subsequent production of TNF-α and IL-6 via the MAPK signaling pathway (Zhang et al., 2016). In turn, microglia-derived IL-6 could induce expression of surface TLR2 and TLR4 and consequent release of cytokines by mast cells, which recruit immune cells (including microglia) to the injured areas (Pietrzak et al., 2011; Aguirre et al., 2013). Conceivably, these two cell types form a feedback loop in concert to promote neuroinflammation.

### Activation of Microglia and Imbalance of Iron Metabolism

Importantly, disturbance of iron homeostasis is a cardinal feature and contributes to the pathogenesis of multiple neurodegenerative diseases including PD, and microglia are capable of modulating the neuronal iron metabolism (Song et al., 2018). Iron is essential for oxygen transport and the related metabolic activities, especially for brain, the highest oxygen-consuming organ. In addition, iron is necessary for various activities exclusively in the brain, such as the maintenance of myelin, the synthesis and metabolism of neurotransmitters including serotonin, norepinephrine and DA (Ward et al., 2014; Crielaard et al., 2017). Iron accumulation is a physiological feature of aging brain, the degree of which seems to be even greater in PD (Ward et al., 2014). The first observation of abnormal deposits of iron in post-mortem brain especially in the basal ganglia of one PD patient was reported by Lhermitte et al. (1924) in 1924, and was later verified by pathological studies (Sofic et al., 1988; Riederer et al., 1989), magnetic resonance imaging studies (Zhang J. et al., 2010; Wang C. et al., 2013; Lehericy et al., 2014) and transcranial ultrasound (Becker and Berg, 2001; Zecca et al., 2005). However, there has long been a debate as to whether iron is a cause or a consequence of this disorder. Abnormal iron accumulation can induce oxidative stress via ROS, especially hydroxyl radical production, leading to the oxidation and modification of proteins, carbohydrates, lipids, and DNA (Ward et al., 2014). Loss of transferrin receptor 1 (involved in iron uptake) rather than ferroportin could cause age-progressive degeneration of dopaminergic neurons and motor deficits in mice (Matak et al., 2016). Iron might modulate the expression of α-syn and facilitate its aggregation and toxicity via oxidative stress-induced nitration and phosphorylation, contributing to degeneration of dopaminergic neurons (Song et al., 2018). Furthermore, neurodegeneration in the injured regions could be exacerbated by the strong redox coupling of iron and DA (Hare and Double, 2016). Regarding microglial contributions to the disturbance of iron metabolism in PD, the expressed transporters/molecules and the released factors such as pro-inflammatory factors, neurotropic factors and lactoferrin, exert fundamental roles (Song et al., 2018). Microglia are more efficient to accumulate and safely store abundant iron than astrocytes and neurons (Bishop et al., 2011), as evidenced by the finding that ferritin was abundant in microglia while rarely detected in astrocytes and neurons (Healy et al., 2016). Even in the case of acute oxidative stress-induced ferritin reduction, the increased iron storage capacity of microglia might be achieved by elevated iron saturation of the existing ferritin molecules (Mehlhase et al., 2005), coupled with the aggregation of activated microglia around degenerative neurons. Further, pro-inflammatory factors released by activated microglia (e.g., IL-1β, IL-6, and TNF-α) contribute to the iron deposition by up-regulating the expression of IRP1 and iron transporters (e.g., DMT1) through production of ROS and RNS (Urrutia et al., 2013; Wang J. et al., 2013). These findings provide compelling evidence that microglia could aggravate iron-mediated neurodegeneration in PD. Understanding iron metabolism and roles of microglia in iron dyshomeostasis might provide new therapeutic strategies for PD via restoring brain iron homeostasis.

### Microbiome-Gut-Brain Axis

In the last decade, the microbiome-gut-brain axis in the pathogenesis of neurodegenerative disorders has attracted increasingly more attention. The gut microbiota could affect BBB permeability (Braniste et al., 2014), and microbiome-derived SCFAs were involved in regulation of microglia homeostasis (Erny et al., 2015). Further, a multi-faceted TLR signaling network is essential for the immune-mediated processes both in the gut and in the CNS, exerting inflammatory or regulatory effects in the pathogenesis of PD (Houser and Tansey, 2017; Caputi and Giron, 2018). Dietary tryptophan metabolites produced by symbiotic microbiota could influence microglial activities, TGF-α production, and neuroinflammatory pathology in EAE, a mouse model of MS (Rothhammer et al., 2018). Future investigations should be focused on whether probiotics or antibiotics or dietary protocols can be utilized to modulate microglial function and to alleviate progress of neurodegenerative diseases including PD.

In brief, as far as PD pathogenesis is concerned, microglial activation and microglia-mediated inflammatory responses play essential roles. Further, α-syn-microglia, neuron-glia, astrocyte-microglia, and mast cell-glia cross-talks (Figure 1), as well as the microbiome-gut-brain axis, are considered to be involved in the regulation of these processes.

## Potentials of Microglia as a Therapeutic Target for PD

Drugs are currently available to relieve PD symptoms by limiting the extent of inflammation (Glass et al., 2010), and thus to enhance the life quality of patients. Nevertheless, these drugs are incapable of repairing or regenerating the damaged neurons (Song and Suk, 2017). Given that the balance of M1/M2 polarization states of microglia has been concerned in connection with the pathogenesis of neurodegenerative disorders, including PD, some small molecular compounds have been studied to determine their potentials to modulate the balance and to elucidate the underlying mechanisms.

### Novel Agents or Approaches Targeting the M1 Polarization State

Drugs targeting pro-inflammatory and pro-killing M1 polarization state could potentially help reduce neuronal damage caused by inflammation. These drugs listed in Table 1 can be divided into several categories:

**TABLE 1:** Novel agents or approaches targeting M1 polarization state.

**Target**	**Agent or approach**	**Description**	**Effects**
TLR2	Candesartan cilexetil (Dasu et al., 2009; Daniele et al., 2015)	A drug approved for cerebrovascular diseases	Inhibit TLR2 and TLR4 expression; reverse M1 phenotype activated by α-syn
	Rifampicin and its autoxidation product rifampicin quinone (Acuna et al., 2019)	Antibiotic	Prevent α-syn-induced TLR2- and P2X7-dependent microglial inflammatory responses *in vitro*
	Endurance exercise (Jang et al., 2017; Koo et al., 2017)		Regulate TLR2 and its downstream signaling (MyD88, TRAF6 and TAK-1)
TLR4	TAK-242 or RSLA (Hughes et al., 2019)	Small molecule antagonists of TLR4	Attenuate α-syn-mediated oxidative stress, TNF-α production by microglia and neuronal death *in vitro*
CB2	β-caryophyllene (Javed et al., 2016; Ojha et al., 2016)	A agonist of CB2 receptors	Suppress microglial activation; inhibit pro-inflammatory cytokine expression
	JWH133 (Chung et al., 2016)	A selective CB2 receptor agonist	Alleviate BBB disruption; suppress peripheral immune cell infiltration; inhibit iNOS and pro-inflammatory cytokine production
JAK/STAT or NF-κB signaling	α-asarone (Kim et al., 2015)	A chemical component found in Annonaceae and Araceae species	Inhibit the NF-κB signaling; inhibit pro-inflammatory factor production; reserve anti-inflammatory factor expression; eventually mitigate behavioral anomalies
	Tanshinone I (Wang et al., 2015b)	A bioactive flavonoid	
NADPH oxidase	Apocynin (Choi et al., 2012; Sharma and Nehru, 2016)	Extracted from *Apocynum cannabinum* etc.	Inhibit microglial activation; suppress the generation of ROS, RNS and pro-inflammatory cytokines; promote anti-inflammatory state
	Resveratrol (Zhang F. et al., 2010)	Extracted from reynoutria etc.	
	Diphenyleneiodonium (Wang et al., 2014, 2015d)	A bivalent iodine compound	
Cytokines	DN-TNF	Intranigral lentiviral delivery of DN-TNF (McCoy et al., 2008; Harms et al., 2011); nigral infusion of XENP345 (McCoy et al., 2006); peripheral application of XPro^®^1595 (Barnum et al., 2014)	Alleviate glial activation; attenuate dopaminergic neuron loss; finally mitigate behavioral deficits
GHS-R1a	Ghrelin (Jiang et al., 2008; Moon et al., 2009)	An endogenous ligand for GHS-R1a	Prevent the activation of microglia; inhibit production of pro-inflammatory factors (TNF-α and IL-1β, NO); attenuate dopaminergic neuron loss
unclear	Lenalidomide (Valera et al., 2015)	Drugs approved for other distinct diseases	Inhibit activation of microglia; attenuate production of pro-inflammatory cytokines
	Zonisamide (Hossain et al., 2018)		
	Minocycline (Wu et al., 2002; Kobayashi et al., 2013)		
	Dimethyl fumarate (Campolo et al., 2017)		
unclear	Ginsenoside Rg1 (Zhou et al., 2015; Heng et al., 2016)	Bioactive compounds extracted from various plants	Inhibit activation of microglia; attenuate production of pro-inflammatory cytokines
	Piperine (Yang et al., 2015)		
	Curcumin (Ghasemi et al., 2019)		
	Rosmarinic acid (Lv et al., 2019)		
	Astilbin (Zhu et al., 2019)		
H_4_R	JNJ7777120 (Zhou et al., 2019)	H_4_R antagonist	Suppress pro-inflammatory activation; prevent dopaminergic neuron degeneration; reduce Lewy body-like neuropathology; improve Parkinson-like behavior
NLRP3	MCC950 (Gordon et al., 2018)	A small-molecule inhibitor of NLRP3 inflammasome	Inhibit NLRP3 inflammasome activation; prevent dopaminergic degeneration and α-syn pathology; alleviate motor behaviors

Firstly, regulation or modification of receptors on microglia, such as TLRs and CB2 receptors, provides a novel pharmacological target for neurodegenerative disorders including PD (Maresz et al., 2005; Cassano et al., 2017; Fiebich et al., 2018; Navarro et al., 2018). In patients with PD, stage and region-dependent TLR2 expression is consistent with region-specific polarization states of microglia, indicating that TLR2 plays a critical role in the microglia-associated responses in this disorder (Doorn et al., 2014). Candesartan cilexetil, an FDA-approved drug for treating cerebrovascular diseases, hypertension, and chronic heart failure, also able to inhibit the expression of TLR2 and TLR4 (Dasu et al., 2009), might reverse the pro-inflammatory phenotype of microglia activated by oligomeric α-syn (Daniele et al., 2015). Moreover, one recent study showed that the antibiotic rifampin and its autoxidation product rifampicin quinone prevented α-syn-induced TLR2- and P2X7-dependent microglial inflammatory responses *in vitro* (Acuna et al., 2019), which needs further investigation to confirm their potentials as anti-parkinsionian drugs. Interestingly, endurance exercise could exert neuroprotective effects in MPTP-induced PD mice by regulating the expression of TLR2 and its downstream signaling such as MyD88, TRAF6, and TAK1 (Jang et al., 2017; Koo et al., 2017). As previously discussed, TLR4, another receptor involved in microglial activation, is a potential therapeutic target. Hughes et al. found that pretreatment with TAK-242 or RSLA, small molecule antagonists of TLR4, significantly attenuated α-syn-mediated oxidative stress, TNF-α production by microglia and neuronal death *in vitro* (Hughes et al., 2019), but has not been tested *in vivo*. The endocannabinoid system is considered to be closely associated with the neuropathological processes of PD (Navarrete et al., 2018). A naturally occurring agonist of CB2 receptors, β-caryophyllene, could negatively regulate microglial activation and suppress expressions of pro-inflammatory cytokines, whereby eliciting significant neuroprotective effects on nigral dopaminergic neurons (Javed et al., 2016; Ojha et al., 2016). JWH133, another selective CB2 receptor agonist, could protect the MPTP induced PD mice by alleviating BBB disruption and by suppressing the peripheral immune cell infiltration as well as by reducing the M1 microglia-production of iNOS and pro-inflammatory cytokines (Chung et al., 2016).

Secondly, the JAK/STAT signaling pathway and the NF-κB signaling pathway are vital for the polarization of microglia to the M1 state. Targeting these signaling pathways may serve as a potential to regulate M1 activation. For example, α-asarone, a chemical component found in various plants (e.g., Annonaceae and Araceae species), was shown to significantly suppress M1 polarization and to attenuate the production of pro-inflammatory cytokines by inhibiting the NF-κB signaling, and to eventually mitigate PD-like behavioral anomalies in the MPTP-induced mouse model (Kim et al., 2015). Tanshinone I, a bioactive flavonoid, could selectively inhibit the activation of NF-κB signaling and the production of M1-pro-inflammatory molecules including IL-1β, IL-6, TNF-α and NO, partially reserve the expression of M2-anti-inflammatory markers such as IL-10, and prevent nigrostriatal dopaminergic neurodegeneration (Wang et al., 2015b).

Thirdly, some compounds targeting NADPH oxidase, such as apocynin, resveratrol and diphenyleneiodonium, could inhibit microglial activation, suppress the generation of various toxic factors (e.g., ROS, RNS, and pro-inflammatory cytokines), and promote microglial phenotype toward anti-inflammatory state, thereby offering promising candidates for clinical trials in patients with neurodegenerative diseases including PD and AD (Zhang F. et al., 2010; Choi et al., 2012; Wang et al., 2014, 2015d; Sharma and Nehru, 2016).

Finally, targeting pro-inflammatory cytokines (e.g., IL-1β and TNF-α) may provide new possibilities to inhibit M1 polarization. In mouse models induced by MPTP or 6-OHDA, selective suppression of solTNF via intranigral lentiviral delivery of DN-TNF (McCoy et al., 2008; Harms et al., 2011) or nigral infusion of DN-TNF compound XENP345 (McCoy et al., 2006) or peripheral application of DN-TNF inhibitor XPro1595 (Barnum et al., 2014) could alleviate glial activation, dopaminergic neuron loss and final behavioral deficits, suggesting that blocking the solTNF signaling by DN-TNF gene transfer or novel agents is therapeutically feasible for PD.

Interestingly, ghrelin, an endogenous ligand for growth hormone secretagogue receptor 1a, was demonstrated to prevent the activation of microglia, the production of pro-inflammatory factors (e.g., TNF-α and IL-1β) and the activation of iNOS, and to attenuate dopaminergic neuron loss in the MPTP-induced mouse models of PD (Jiang et al., 2008; Moon et al., 2009). The inhibitory effect of ghrelin seems to be exerted by suppressing the expression of MMP-3 in stressed dopaminergic neurons (Moon et al., 2009).

In addition, several drugs approved for other distinct diseases [e.g., lenalidomide for multiple myeloma or myelodysplastic syndrome (Valera et al., 2015), zonisamide for epilepsy (Hossain et al., 2018), minocycline for infectious diseases (Wu et al., 2002; Kobayashi et al., 2013), dimethyl fumaratefor MS (Campolo et al., 2017)] and some bioactive compounds [e.g., ginsenoside Rg1 (Zhou et al., 2015; Heng et al., 2016), piperine (Yang et al., 2015), curcumin (Ghasemi et al., 2019), rosmarinic acid (Lv et al., 2019), astilbin (Zhu et al., 2019)] are capable of inhibiting activation of microglia and attenuating production of pro-inflammatory cytokines in mouse models of PD. These drugs might offer novel alternative therapeutic approaches for PD. However, the underlying mechanisms or involved signaling pathways are not fully understood and require further investigations.

Several promising targets have been proposed to modulate microglial activation. For example, microglia-mediated immune responses could be enhanced by CD73-derived adenosine A2A signaling. CD73 inactivation substantially limited adenosine production, inhibited A2A receptor (A2AR)-mediated pro-inflammatory state of microglia, enhanced the activity of dopaminergic neurons, and mitigated the motor symptoms in animal models of PD (Meng et al., 2019). Histamine-4 receptor is another regulator of microglial activation. Lateral ventricle infusion of H_4_R antagonist JNJ7777120 could suppress the pro-inflammatory activation of microglia, prevent the degeneration of dopaminergic neurons, reduce the Lewy body-like neuropathology, and improve parkinsonian behaviors (Zhou et al., 2019). Another potential target for PD, NLRP3 inflammasome in microglia can be activated by fibrillar α-syn (Gordon et al., 2018). Oral administration of a small-molecule inhibitor of NLRP3 inflammasome, MCC950, effectively prevented dopaminergic degeneration and α-syn pathology, and alleviated motor behaviors in PD models (Gordon et al., 2018).

### Novel Agents or Approaches Capable of Enhancing the M2 Phenotype or Induce the Phenotypic Switch From M1 to M2

Some molecules (e.g., IL-10, cyclic AMP, and vitamin D), which have the capability of enhancing the anti-inflammatory phenotype or of inducing the phenotypic switch from pro-inflammatory to anti-inflammatory, may have therapeutic feasibility for PD (Table 2).

**TABLE 2:** Novel agents or approaches capable of enhancing M2 phenotype or induce the phenotypic switch from M1 to M2.

**Key molecule**	**Agent or approach**	**Mechanism**	**Effects**
IL-10	Intracerebral injection of AAV2-hIL-10 (Schwenkgrub et al., 2013; Joniec-Maciejak et al., 2014)	IL-10 is capable of suppressing iNOS expression via the inhibition of NF-κB activity (Schwenkgrub et al., 2013).	Enhance tyrosine hydroxylase protein expression; inhibit iNOS expression; promote anti-inflammatory factor production (e.g., TGF-β and IFN-γ)
Cyclic AMP	PDE4 inhibitor rolipram (Kinoshita et al., 2017)	PDE inhibitors elevate production of cyclic AMP; cyclic AMP is considered to be a key intracellular regulator of microglial phenotypic conversion in the presence of IL-4 (Ghosh et al., 2016).	Abate production of oxidative molecules (e.g., ROS) and pro-inflammatory factors (e.g., TNF-α and IP-10); improve phagocytic properties
	PDE5 inhibitors sildenafil and yonkenafil (Zhao et al., 2016)		
	Non-selective PDE inhibitor ibudilast (Schwenkgrub et al., 2017)		
Vitamin D (Kim et al., 2006; Dulla et al., 2016; Verma and Kim, 2016; Calvello et al., 2017)		Vitamin D is capable of inhibiting microglial activation, shifting M1 to M2 polarized states via suppression of ERK activation (Dulla et al., 2016) and an IL-10 dependent SOCS3 mechanism (Boontanrart et al., 2016).	Attenuate M1 with decreased iNOS and TLR4 expression and reduced pro-inflammatory factor production (IL-1β, IL-6, IL-12, TNF-α, and NO); facilitate M2 with elevated TLR10 expression and increased anti-inflammatory mediator generation (IL-4, IL-10, TGF-β, and CCL17)
PPAR-γ agonists	Pioglitazone (Dehmer et al., 2004; Swanson et al., 2011)	PPARs play vital roles in the down-regulation of microglial activation, neuroinflammation, oxidative stress, proteasomal dysfunction, and mitochondrial dysfunction via regulation of CD200 and CD200R1 expression (Dentesano et al., 2014).	Act via activation of PPAR-γ, induction of IκBα, block of NF-κB activation, iNOS induction and NO-mediated toxicity; shift M1 to M2; improve motor symptoms
	Rosiglitazone (Pisanu et al., 2014)		
	An N-carbamolylated urethane compound SNU-BP (Song et al., 2016)		

IL-10 is a potent anti-inflammatory cytokine capable of suppressing iNOS expression via the inhibition of the NF-κB activity (Schwenkgrub et al., 2013). Several studies have demonstrated that intracerebral injection of AAV2-hIL-10 in MPTP-induced mouse models of PD could enhance the expression of the striatal tyrosine hydroxylase protein associated with down-regulated expressions of pro-inflammatory iNOS, and promote the production of anti-inflammatory factors like TGF-β, suggesting the neuroprotective properties of AAV2-hIL-10 (Schwenkgrub et al., 2013; Joniec-Maciejak et al., 2014).

Cyclic AMP is considered to be a key intracellular regulator of microglial phenotypic conversion (Ghosh et al., 2016). The M1 to M2a conversion could be promoted by cyclic AMP combined with IL-4, but neither alone, in *in vitro* microglia cell culture. The M2a-converted microglia showed abated production of oxidative molecules (e.g., ROS) and pro-inflammatory factors (e.g., TNF-α and IP-10), as well as improved phagocytic properties. The cyclic AMP-protein kinase A signaling pathway was involved in the conversion process (Ghosh et al., 2016). Based on this finding, application of synthetic analogs of cyclic AMP, activators of adenylyl cyclase, or inhibitors of PDE might suppress the pro-inflammatory phenotype of microglial cells via elevation of cyclic AMP. Notably, PDE inhibitors, such as rolipram for PDE4, sildenafil and yonkenafil for PDE5 (Zhao et al., 2016), and non-selective PDE inhibitors (e.g., ibudilast), have been proposed as potential therapeutic drugs for neurodegenerative disorders, including MS (Pifarre et al., 2011), AD (Myeku et al., 2016; Kumar and Singh, 2017; Rabal et al., 2018; Xu et al., 2018), and PD (Kinoshita et al., 2017; Schwenkgrub et al., 2017).

Vitamin D is capable of protecting dopaminergic neurons against neuroinflammation and oxidative stress via inhibition of microglial activation, shifting M1 to M2 polarized states, which has been demonstrated in mouse models of PD induced by 6-OHDA/MPTP (Kim et al., 2006; Calvello et al., 2017; Lima et al., 2018) and in microglial HMO6/BV-2 cells (Boontanrart et al., 2016; Dulla et al., 2016; Verma and Kim, 2016). Vitamin D treatment could attenuate M1 polarization with decreased expression of iNOS and TLR4 and reduced production of pro-inflammatory factors (IL-1β, IL-6, IL-12, TNF-α, and NO), and facilitate M2 polarization with elevated expression of TLR10 and increased generation of anti-inflammatory mediators (IL-4, IL-10, TGF-β, and chemokine CCL17) and typical hallmarks of M2 microglia (CD163, CD204, and CD206) (Kim et al., 2006; Dulla et al., 2016; Verma and Kim, 2016; Calvello et al., 2017). Regulatory effects of vitamin D on microglial activation might be associated with suppression of ERK activation (Dulla et al., 2016) and an IL-10 dependent SOCS3 mechanism (Boontanrart et al., 2016).

Peroxisome proliferator-activated receptors, nuclear receptors involved in the pathogenesis of neurodegenerative disorders, have garnered much attention as a promising therapeutic target. PPARs down-regulate microglial activation, neuroinflammation and oxidative stress (Agarwal et al., 2017). PPAR-γ agonists, such as pioglitazone and rosiglitazone, could modulate microglial polarization, thus playing neuroprotective roles on dopaminergic neurons. Oral administration of pioglitazone improved motor symptoms of parkinsonian rhesus monkeys induced by MPTP via anti-inflammatory and neuroprotective effects (Swanson et al., 2011). Pioglitazone might sequentially act via activation of PPAR-γ, induction of IκBα, blockade of NF-κB activation, iNOS induction and NO-mediated toxicity in an MPTP-induced mouse model of PD (Dehmer et al., 2004). In addition, an *in vitro* study indicated that the anti-inflammatory and neuroprotective effects of PPAR-γ agonists might be closely associated with the interaction between CD200 and CD200R1 (Dentesano et al., 2014). Similarly, rosiglitazone could boost the M2 (anti-inflammatory) phenotype, and regulate cytokine production, whereby alleviating the degeneration of dopaminergic neurons in both SNpc and striatum (Pisanu et al., 2014). However, clinical studies as to whether glitazone protected against PD yielded controversial results (Brauer et al., 2015; Connolly et al., 2015; Ninds Exploratory Trials in Parkinson Disease (Net-Pd) Fs-Zone Investigators., 2015; Brakedal et al., 2017). Notably, a phase 2, multi-center, double-blind, placebo-controlled, randomized clinical trial reported that pioglitazone seemed incapable of modifying progression in early PD (Ninds Exploratory Trials in Parkinson Disease (Net-Pd) Fs-Zone Investigators., 2015). Some novel PPAR-γ agonists, such as an N-carbamylated urethane compound SNU-BP (Song et al., 2016), may inhibit M1-pro-inflammatory cytokine and NO production, and enhance M2 marker expression. Further studies are still needed to confirm the therapeutic potentials of PPAR agonists in neurodegeneration and to explore the underlying mechanisms.

In summary, a variety of drugs may play neuroprotective roles in PD by regulating microglial activation and microglia-induced inflammation; nevertheless, the therapeutic mechanisms are not fully understood (Tables 1, 2). In view of roles of iron metabolism in PD pathogenesis, the protective effects of iron chelation has been explored in the MPTP mouse model by both genetic (transgenic expression of the iron binding protein ferritin) and pharmacological (oral administration of the bioavailable metal chelator clioquinol) means (Kaur et al., 2003). Deferiprone, another oral iron chelator used to treat systemic iron overload diseases, was demonstrated to increase hippocampal BDNF levels, rescue memory deficits and ameliorate iron-induced harmful effects (Alcalde et al., 2018). Further investigations are warranted to explore the underlying mechanisms of beneficial effects of these drugs and to discover other novel potentially therapeutic approaches. It is noteworthy, however, that the pharmacological modulation of microglia could also have adverse effects as microglia have such physiological functions mentioned aboved. One recent study found that blockade of CD22, a canonical B-cell receptor which is up-regulated on aged microglia, reprogramed microglia toward a homeostatic transcriptional state and restored homeostatic microglial phagocytosis of α-syn fibrils (Pluvinage et al., 2019).

Apart from these pharmacological treatments, rTMS have been reported to have modulating effect (promoting/suppressing) on microglial activation, which may depend on the intensity and frequency of rTMS (Cullen and Young, 2016; Sasso et al., 2016; Cacace et al., 2017). Further research are needed to clarify the specific effect of rTMS on microglia as well as on astrocytes and neurons. In addition, accumulating evidence indicates that physical exercise could inhibit microglial activation and regulate neuroinflammation as well as improve the synaptic plasticity, whereby enhancing brain function and attenuating neurodegeneration (Mee-Inta et al., 2019).

## Future Perspectives

Accumulating studies on PD pathogenesis have focused on activation of microglia and cross-talks between microglia and other immune factors. Activated microglia bear pro-inflammatory or anti-inflammatory properties depending on the nature, intensity and duration of the stimulus and associated with disease stage and severity. Importantly, activated microglia may exist along a dynamic continuum rather than be simply polarized into two categories. Activation of microglia triggered by damaged dopaminergic neurons could cause neuroinflammation, oxidative stress and cytokine-receptor-mediated apoptosis, which in turn enhance peripheral leucocyte recruitment. In this way, a vicious cycle comes into being, resulting in the exacerbation of the neurodegenerative process. In addition, cross-talks of α-syn-microglia, neuron-glia, astrocyte-microglia, and mast cell-glia as well as the microbiome-gut-brain axis are thought to be involved in the regulation of microglial activation and neuroinflammation. The existing available drugs of PD are able to mitigate symptoms by limiting the extent of inflammation (Glass et al., 2010). Remarkably, the strategy of the biological therapy should be to restore the immune balance, rather than simply inhibit the immune responses. A variety of agents or approaches, including candesartan cilexetil, rifampicin/rifampicin quinone, TAK-242, RSLA, β-caryophyllene, JWH133, α-asarone, tanshinone I, apocynin, resveratrol, diphenyleneiodonium, DN-TNF, lenalidomide, zonisamide, minocycline, dimethyl fumarate, some bioactive compounds extracted from various plants, JNJ7777120, MCC950, AAV2-hIL-10, Vitamin D, PDE inhibitors (rolipram, ibudilast), and PPAR-γ agonists (pioglitazone, rosiglitazone), might exert neuroprotective effects due to their regulatory effects on microglial activation. In addition, intervention of iron metabolism balance or microbiome-gut-brain axis as well as rTMS and physical exercise may offer emerging therapeutic strategies. In conclusion, microglial activation is a key point to understand the pathogenesis of PD and a potential therapeutic target for PD. Further investigations should focus on the dual role of reactive microglia to elucidate PD pathogenesis and detect emerging agents/approaches for PD.

## Author Contributions

C-YL, XW, and CL searched the literature and drafted the manuscript. C-YL and H-LZ critically revised the manuscript. All authors listed have made a substantial, direct, and intellectual contribution to the work, and approved it for publication.

## Conflict of Interest

The authors declare that the research was conducted in the absence of any commercial or financial relationships that could be construed as a potential conflict of interest.

## Abbreviations

α-syn: α-synuclein
6-OHDA: 6-hydroxydopamine
AAV2-hIL-10: adeno-associated viral vector containing the cDNA for human interleukin-10
AD: Alzheimer’s disease
BBB: blood-brain barrier
BDNF: brain-derived neurotrophic factor
bFGF: basic fibroblast growth factor
CB2: cannabinoid type 2
CNS: central nervous system
COX: cyclooxygenase
CX3CR1: CX3C chemokine receptor 1
CXCL: chemokine (C-X-C motif) ligand
cyclic AMP: cyclic adenosine monophosphate
DA: dopamine
DAP12: DNAX activation protein 12
DMT1: divalent metal transporter 1
DN-TNF: dominant-negative tumor necrosis factor
EAA: excitatory amino acids
EAE: experimental autoimmune encephalomyelitis
EGF: epidermal growth factor
ERK: extracellular signal-regulated kinase
GSH: glutathione
GWAS: genome-wide association studies
H_4_R: histamine-4 receptor
HLA: human leucocyte antigen
I κ B α: inhibitory protein- κ-Bα
IFN: interferon
IGF-1: insulin-like growth factor-1
IKK: I κ B kinase
IL: interleukin
iNOS: inducible nitric oxide synthase
IP-10: interferon-gamma-induced protein-10
IRAK: interleukin-1 receptor-associated kinase
IRF: interferon regulatory factor
IRP1: iron regulatory protein 1
JAK: Janus kinase
LPS: lipopolysaccharide
MAPK: mitogen-activated protein kinase
MHC: major histocompatibility complex
MMP: matrix metalloproteinase
MPTP: 1-methyl-4-phenyl-1,2,3,6-tetrahydropyridine
MS: multiple sclerosis
NADPH: nicotinamide adenine dinucleotide phosphate
NF- κ B: nuclear factor kappa B
NGF: nerve growth factor
NLRP3: NLR family pyrin domain containing 3
NO: nitric oxide
NSAID: nonsteroidal anti-inflammatory drug
NSPC: neural stem/progenitor cell
PAR-1: protease-activated receptor-1
PARP: poly ADP ribose polymerase
PD: Parkinson’s disease
PDE: phosphodiesterase
PET: positron emission tomography
PG: prostaglandins
PPAR: peroxisome proliferator-activated receptor
P-SAPK/JNK: phospho-stress-activated protein kinases/c-Jun N-terminal kinases
RNS: reactive nitrogen species
ROS: reactive oxygen species
rTMS: repetitive transcranial magnetic stimulation
SCFAs: short-chain fatty acids
SN: substantia nigra
SNpc: substantia nigra (SN) pars compacta
SOCS3: suppressor of cytokine signaling 3
solTNF: soluble tumor necrosis factor
SR: scavenger receptor
STAT: signal transducer and activator of transcription
TAK1: TGF- β-activated protein kinase 1
Tem: effector/memory T cells
TGF: transforming growth factor
TIR: toll-interleukin 1 receptor
TLR: toll-like receptor
TNF: tumor necrosis factor
TRAF6: TNF receptor-associated factor 6
TREM2: triggering receptor expressed on myeloid cells 2
TRPC1: transient receptor potential canonical 1
UPDRS-III: Unified Parkinson’s Disease Rating Scale III
